# Metabolic Syndrome Modulates Association between Endothelial Lipase and Lipid/Lipoprotein Plasma Levels in Acute Heart Failure Patients

**DOI:** 10.1038/s41598-017-01367-2

**Published:** 2017-04-26

**Authors:** Ines Potočnjak, Matias Trbušić, Sanda Dokoza Terešak, Bojana Radulović, Gudrun Pregartner, Andrea Berghold, Beate Tiran, Gunther Marsche, Vesna Degoricija, Saša Frank

**Affiliations:** 1 University Hospital Centre Sisters of Charity, Department of Medicine, Zagreb, Croatia; 2 University of Zagreb School of Medicine, Zagreb, Croatia; 3 University Hospital Centre Zagreb, Zagreb, Croatia; 4 Institute for Medical Informatics, Statistics und Documentation, Medical University of Graz, Graz, Austria; 5 Clinical Institute for Medical and Chemical Laboratory Diagnostics, Medical University of Graz, Graz, Austria; 6 Institute of Experimental and Clinical Pharmacology, Medical University of Graz, Graz, Austria; 7 Institute of Molecular Biology and Biochemistry, Centre of Molecular Medicine, Medical University Graz, Graz, Austria

## Abstract

We hypothesised that the established association of endothelial lipase (EL) plasma levels with atherogenic lipid profile is altered in acute heart failure (AHF) and additionally affected by overlapping metabolic syndrome (MetS). We examined the association of EL plasma levels and lipid/lipoprotein plasma levels in AHF patients without and with overlapping MetS. The study was performed as a single-centre, observational study on 152 AHF patients, out of which 85 had overlapping MetS. In the no-MetS group, EL plasma levels were significantly positively correlated with plasma levels of atherogenic lipids/lipoproteins, including total cholesterol, low-density lipoprotein (LDL)-cholesterol, total LDL particles and triglycerides, but also with plasma levels of antiatherogenic high-density lipoprotein (HDL)-cholesterol, total HDL particles and small HDL particles. In the MetS group, EL plasma levels were positively correlated with triglyceride and small LDL-particle levels, and significantly negatively correlated with plasma levels of large HDL particles as well as with LDL- and HDL-particle size, respectively. EL- and lipid/lipoprotein- plasma levels were different in the no-MetS patients, compared to MetS patients. The association of EL with atherogenic lipid profile is altered in AHF and additionally modified by MetS, which strongly modulates EL- and lipid/lipoprotein-plasma levels in AHF.

## Introduction

Endothelial lipase (EL) is a member of the triglyceride lipase gene family, expressed primarily by vascular endothelial cells (ECs) and to a lesser extent smooth muscle cells (SMCs) and macrophages^1, 2^. EL is a phospholipase which has a high affinity for high-density lipoprotein-phospholipids. EL expression can be induced by tumor necrosis factor-α, interleukin-1β and biomechanical forces in vascular EC^3, 4^, by angiotensin II and hypertension in vascular SMCs^5^ as well as by lipopolysaccharide in macrophages^6^. EL concentrations in human plasma have been found to be strongly associated with inflammatory markers, such as C-reactive protein (CRP), interleukin-6 (IL-6) and secretory phospholipase A2 type IIA levels^7^. EL plasma levels are increased in metabolic syndrome (MetS) and associated with subclinical atherosclerosis, measured as coronary artery calcification^8^. In contrast to EL plasma levels, the EL activity in post-heparin plasma was not significantly increased in humans with MetS, but was dependent on the degree of insulin resistance^9^. EL is expressed in human and mouse atherosclerotic plaques mainly by macrophages and to a lesser extent by SMCs^10–12^. EL modulates HDL plasma levels and functionality^13–16^, and its plasma levels are associated with a proatherogenic lipid profile^8, 17^. Moreover, EL was recognized as an important provider of lipoprotein-derived fatty acids to cardiac tissue of mice with pressure overload-induced heart failure (HF), a pathophysiological condition accompanied by an increased energy demand and a decreased cardiac lipoprotein lipase (LPL) expression; EL deficient mice presented more severe HF than wild type mice due to decreased cardiac uptake of fatty acids^18^.

Decreased lipid synthesis and decreased intestinal lipid absorption due to venous congestion, a consequence of right-sided heart failure, underlie the low lipid plasma levels and deranged lipid metabolism in HF^19, 20^. Considering the disturbed lipid metabolism in HF and metabolic perturbations with upregulation of EL in MetS^8, 21^,we hypothesised that the established association of EL plasma levels and atherogenic lipid profile, including increased triglycerides, total cholesterol and apoB-containing lipoproteins, as well as decreased HDL-cholesterol plasma levels^8, 15–17^, is altered in acute HF (AHF) and additionally affected by overlapping MetS.

## Results

### Patients’ clinical characteristics

Of the152 AHF patients included in the study, 85 (55.9%) had MetS (Table 1). The MetS and the no-MetS groups did not differ significantly regarding gender, age, mean arterial pressure (MAP), New York Heart Association (NYHA) classification, time of AHF onset, EF, smoking and final clinical presentation (Table 1). As obvious from patients’ age none of the groups contained premenopausal women. In line with the MetS definition, the MetS group had significantly higher body weight, body mass index (BMI) and waist circumference, as well as a higher incidence of hypertension, type 2 diabetes mellitus (T2DM), hyperlipidaemia/hypertriglyceridaemia and hypercholesterolaemia (Table 1). Additionally, the MetS group had a higher incidence of chronic obstructive pulmonary disease (COPD) (Table 1). The no-MetS and MetS groups had a similar incidence of enlarged liver, ascites and peripheral oedema, the signs implying venous overload due to right-sided heart failure (Table 1).

**Table 1:** Baseline characteristics, classification, comorbidities and signs at admission.

Variable	no-MetS n = 67 (44.1%)	MetS n = 85 (55.9%)	P-value
**Baseline characteristics**			
Female (n)	41 (61.2%)	38 (44.7%)	0.051
Age (years)	76.6 (10.12)	74.1 (10.28)	0.137
MAP (mmHg)	104.8 (22.64)	106.0 (21.44)	0.739
Body weight (kg)	72.6 (16.5)	91.1 (18.1)	**<0.001**
BMI (kg/m^2^)	25.7 (4.03)	31.3 (5.10)	**<0.001**
Waist circumference (cm)	100.9 (15.13)	119.3 (15.21)	**<0.001**
Smoking	14 (20.9%)	24 (28.2%)	0.348
NYHA Class 2	4 (6.0%)	7 (8.2%)	0.846
NYHA Class 3	38 (56.7%)	45 (52.9%)	
NYHA Class 4	25 (37.3%)	33 (38.8%)	
**Classifications**			
De novo	18 (26.9%)	29 (34.1%)	0.380
Worsening of CHF	49 (73.1%)	56 (65.9%)	
HFrEF	37 (58.7%)	46 (56.8%)	0.866
HFpEF	26 (41.3%)	35 (43.2%)	
**Final Clinical Presentation**			
Worsening of CHF	37 (55.2%)	41 (48.2%)	0.178
Hypertensive AHF	11 (16.4%)	11 (12.9%)	
Isolated Right Side AHF	1 (1.5%)	6 (7.1%)	
ACS and HF	6 (9.0%)	17 (20.0%)	
Pulmonary edema	11 (16.4%)	9 (10.6%)	
CS	1 (1.5%)	1 (1.2%)	
**Comorbidities**			
Atherosclerosis	28 (41.8%)	41 (48.2%)	0.512
CM	47 (70.1%)	66 (77.6%)	0.351
ACS	7 (10.4%)	17 (20.0%)	0.122
Hypertension	55 (82.1%)	81 (95.3%)	**0.014**
T2DM	17 (25.8%)	61 (71.8%)	**<0.001**
Hyperlipidaemia	19 (28.4%)	41 (48.2%)	**0.019**
Hypercholesterolaemia	20 (29.9%)	39 (45.9%)	**0.047**
COPD	12 (17.9%)	29 (34.1%)	**0.028**
CKD	24 (35.8%)	26 (30.6%)	0.602
Anemia	17 (25.4%)	22 (25.9%)	1.000
**Signs at admission**			
Enlarged liver	24 (35.8%)	29 (34.1%)	0.865
Ascites	6 (9.0%)	15 (17.6%)	0.157
Peripheral edema	45 (67.2%)	60 (70.6%)	0.725
Any single sign or combination thereof	49 (73.1%)	61 (71.8%)	1.000

Metric/continuous/quantitative variables are presented as mean and standard deviation. For categorical/qualitative variables the absolute and relative frequencies are shown as n (%) and significant differences depicted in bold.

ACS-Acute Coronary Syndrome; AHF-Acute Heart Failure; BMI-Body Mass Index; BW-Body Weight; CHF-Chronic Heart Failure; CKD-Chronic Kidney Disease, CM-Cardiomyopathy; COPD-Chronic Obstructive Pulmonary Disease; CS-Cardiogenic Shock; EF-Ejection Fraction; HFpEF-Heart Failure with preserved Ejection Fraction; MAP-Mean arterial pressure; HFrEF- Heart Failure with reduced Ejection Fraction; NYHA-New York Heart Association Functional Classification; T2DM-Type 2 Diabetes Mellitus.

### Laboratory parameters

In accordance with previous reports^8, 17^, EL plasma levels were higher in the MetS group compared with the no-MetS group (Table 2). Total cholesterol and low-density lipoprotein (LDL)-cholesterol were similar in the MetS and the no-MetS groups (Table 2). In line with the MetS definition, the triglyceride plasma levels were significantly higher, and HDL-cholesterol plasma levels were significantly lower in the MetS compared to the no-MetS group (Table 2). Additionally, we found significantly higher plasma levels of large very low-density lipoprotein particles (LVLDL-p) and significantly lower plasma levels of large HDL particles (LHDL-p) in the MetS compared with the no-MetS group (Table 2). Furthermore, the size of HDL particles (HDL-s) was significantly smaller in the MetS compared with the no-MetS group (Table 2). The concentrations of CRP and interleukin-6 (IL-6) were similar in both groups (Table 2).

**Table 2:** Laboratory parameters.

Variable	n	no-MetS	n	MetS	P-value
EL (pg/mL)	66	383.1 [74.1–1013.4]	82	462.3 [157.6–1407.7]	**0.013**
IL-6 (pg/mL)	66	18.7 [0.4–300.0]	82	21.0 [1.2–300.0]	0.483
CRP (µg/mL)	66	9.0 [0.2–169.0]	85	10.6 [0.8–247.4]	0.347
Total cholesterol (mmol/L)	66	3.8 [2.2–9.1]	85	3.8 [1.7–7.7]	0.672
LDL-cholesterol (mmol/L)	67	2.3 [0.8–6.3]	85	2.3 [1.0–6.0]	0.281
HDL-cholesterol (mmol/L)	67	1.1 [0.5–2.3]	85	0.9 [0.3–3.6]	**0.004**
Triglycerides (mmol/L)	67	1.0 [0.5–3.0]	85	1.2 [0.6–4.3]	**0.001**
LVLDL-p (nmol/L)	29	1.9 [1.5–5.1]	47	2.7 [1.5–10.6]	**<0.001**
TLDL-p (nmol/L)	60	1100.5 [564–2488]	75	1097 [360–2425]	0.856
LLDL-p (nmol/L)	59	684 [304–2078]	67	689 [300–1160]	0.839
SLDL-p (nmol/L)	58	451 [176–1440]	74	473.5 [172–1331]	0.150
THDL-p (nmol/L)	60	21924 [3711–37506]	75	20774 [7910–36951]	0.713
LHDL-p (nmol/L)	53	6304 [2943–14726]	55	4761 [2829–14921]	**<0.001**
SHDL-p (nmol/L)	48	17261 [7086–32390]	67	16502 [6332–34210]	0.858
VLDL-s (nm)	60	47.1 [39.4–52.3]	75	46.9 [37.1–51.3]	0.968
LDL-s (nm)	60	21.3 [20.7–22.7]	75	21.2 [20.2–21.9]	0.084
HDL-s (nm)	60	9.4 [8.5–10.5]	75	9.0 [8.3–10.6]	**<0.001**

Results are presented as median, minimum and maximum. Significant differences between MetS and noMetS patients were calculated with Mann-Whitney U-test and are depicted in bold. HDL-high density lipoprotein; LDL-low-density lipoprotein; IL-6-interleukin 6; hsCRP-C-reactive protein; EL-endothelial lipase; LVLDL-p –large very low-density lipoprotein particles; TLDL-p –total LDL particles; LLDL-p –large LDL particles; SLDL-p –small LDL-p; THDL-p –total HDL-particles; LHDL-p –large HDL-p; SHDL-p –small HDL-p; VLDL-s –VLDL size; LDL-s –LDL size; HDL-s –HDL size; n- number of samples in which particular parameter was analysed.

Reference values: Triglycerides (mmol/L) 0.5–1.8 (male); 0.5–1.5 (female); total cholesterol (mmol/L) 3.5–5.2; HDL-cholesterol (mmol/L) ≥1.4 (male); ≥1.7 (female); LDL-cholesterol (mmol/L) 2.0–3.9. ^a^SI measurements. To convert to mg/dL, multiply Triglycerides by 89 and total cholesterol, HDL-cholesterol or LDL-cholesterol by 39.

### Correlation of EL plasma levels and plasma lipids/lipoproteins

In the no-MetS group, EL plasma levels were significantly positively correlated with total cholesterol, LDL-cholesterol, HDL-cholesterol, triglycerides, total low-density lipoprotein particle concentrations (TLDL-p), total (T) HDL-p and small (S) HDL-p (Table 3). In the MetS group, similarly to the no-MetS group, EL plasma levels were positively correlated with triglyceride levels (Table 3). In contrast to the no-MetS group, in the MetS group EL was significantly positively correlated with small (S)LDL-particles and significantly negatively correlated with plasma levels of LHDL-particles as well as with LDL-s and HDL-s (Table 3). As shown by partial correlation analysis, T2DM did not affect the correlation between EL and lipids/lipoproteins (Supplementary Table S1).

**Table 3:** Spearman correlation of EL plasma levels with plasma lipids and lipoproteins.

	no-MetS		MetS	
	rho	p-value	rho	p-value
Total cholesterol (mmol/L)	0.46	**<0.001**	0.19	0.090
LDL-cholesterol (mmol/L)	0.38	**0.002**	0.16	0.153
HDL-cholesterol (mmol/L)	0.45	**<0.001**	−0.11	0.318
Triglycerides (mmol/L)	0.30	**0.013**	0.29	**0.007**
LVLDL-p (nmol/L)	0.21	0.272	0.12	0.430
TLDL-p (nmol/L)	0.28	**0.029**	0.16	0.161
LLDL-p (mmol/L)	0.24	0.071	−0.02	0.857
SLDL-p (nmol/L)	0.06	0.650	0.24	**0.042**
THDL-p (nmol/L)	0.47	**<0.001**	0.17	0.153
LHDL-p (nmol/L)	0.13	0.348	−0.38	**0.004**
SHDL-p (nmol/L)	0.34	**0.019**	0.22	0.076
VLDL-s (nm)	0.14	0.286	−0.12	0.304
LDL-s (nm)	0.10	0.457	−0.24	**0.036**
HDL-s (nm)	−0.12	0.352	−0.32	**0.006**

HDL-high density lipoprotein; LDL-low-density lipoprotein; LVLDL-p –large very low-density lipoprotein particle; TLDL-p –total LDL particles; LLDL-p –large LDL particles; SLDL-p –small LDL-p; THDL-p –total HDL-particles; LHDL-p –large HDL-p; SHDL-p –small HDL-p; VLDL-s –VLDL size; LDL-s –LDL size; HDL-s –HDL size. Significant associations are depicted in bold.

### Impact of venous overload on EL and lipid/lipoprotein plasma levels

Considering the role of venous congestion in the deranged lipid metabolism in HF, together with the increased EL and metabolic perturbations in MetS, we examined whether venous volume overload affects EL- and lipid/lipoprotein- plasma levels, and whether the impact of venous volume overload is modulated by MetS. For this purpose we compared EL- and lipid/lipoprotein- plasma levels in the no-MetS and the MetS patients, having none vs. any one or more of the three signs implying volume overload (enlarged liver, peripheral oedema or ascites), as a consequence of right- sided HF. Within the no-MetS group, plasma levels of EL, total cholesterol, LDL-cholesterol, HDL-cholesterol and LLDL-p were significantly decreased in patients with sign(s) implying volume overload (Table 4). This was not observed in the MetS group (Table 4). Triglyceride-, THDL-p- and SHDL-p- plasma levels were significantly decreased in both the no-MetS and MetS groups, and plasma levels of SLDL-p were decreased only in the MetS group with sign(s) implying volume overload, compared to patients with no sign(s) (Table 4).

**Table 4:** EL and lipid/lipoprotein plasma levels in patients without or with sign(s) implying volume overload.

	no-MetS			MetS		
	No Sign(s) n = 18	Sign(s) n = 49	p-value	No Sign(s) n = 24	Sign(s) n = 61	p-value
EL (pg/mL)	504.6 [246.0–1013.4]	362.0 [74.1–913.3]	**0.030**	566.1 [256.7–1407.7]	459.1 [157.6–1387.6]	0.157
Total cholesterol (mmol/L)	5.7 [2.5–9.1]	3.7 [2.2–8.5]	**0.001**	4.2 [2.5–6.4]	3.8 [1.7–7.7]	0.134
LDL-cholesterol (mmol/L)	3.7 [1.4–6.3]	2.2 [0.8–6.1]	**0.008**	2.4 [1.3–4.5]	2.1 [1.0–6.0]	0.117
HDL-cholesterol (mmol/L)	1.4 [0.6–2.3]	1.0 [0.5–1.9]	**0.002**	1.0 [0.4–1.4]	0.9 [0.3–3.6]	0.490
Triglycerides (mmol/L)	1.1 [0.6–2.8]	0.9 [0.5–3.0]	**0.015**	1.5 [0.6–4.3]	1.1 [0.6–3.2]	**0.041**
LVLDL-p (nmol/L)	2.5 [1.5–5.1]	1.7 [1.5–3.7]	0.077	3.0 [1.7–8.3]	2.7 [1.5–10.6]	0.239
TLDL-p (nmol/L)	1530 [564–2488]	1084.5 [615–2437]	0.110	1352.5 [614–2425]	1081.0 [360–2351]	0.091
LLDL-p (nmol/L)	980.5 [312–2078]	625.0 [304–1870]	**0.022**	740.0 [320–1154]	654.0 [300–1160]	0.211
SLDL-p (nmol/L)	410.0 [176–1082]	471.0 [236–1440]	0.073	567.5 [349–1331]	451.0 [172–1191]	**0.030**
THDL-p (nmol/L)	30463 [8741–37506]	19140 [3711–30373]	**<0.001**	26083 [9668–36951]	20050 [7910–36377]	**0.006**
LHDL-p (nmol/L)	6212 [4748–14204]	6317.5 [2943–14726]	0.690	4481.5 [2829–7123]	4761 [2835–14921]	0.231
SHDL-p (nmol/L)	24486.5 [15680–32390]	14918.5 [7086–23044]	**<0.001**	21729 [13532–34210]	15246 [6332–28611]	**0.001**

Results are presented as median, minimum and maximum. Significant differences between patients with no sign(s) and those with one or combination of two or three sign(s) implying volume overload were calculated with Mann-Whitney U-test and are depicted in bold. HDL-high density lipoprotein; LDL-low-density lipoprotein; EL-endothelial lipase; LVLDL-p –large very low-density lipoprotein particles; TLDL-p –total LDL particles; LLDL-p –large LDL particles; SLDL-p –small LDL-p; THDL-p –total HDL-particles; LHDL-p –large HDL-p; SHDL-p –small HDL-p; VLDL-s –VLDL size; LDL-s –LDL size; HDL-s –HDL size.

## Discussion

We hypothesised that the reported association of EL and increased plasma triglycerides, total cholesterol and apoB-containing lipoproteins, as well as with decreased HDL-cholesterol plasma levels^8, 15–17^, is altered in AHF and additionally affected by MetS. To test our hypothesis, we examined the association of EL plasma levels with plasma lipids/lipoproteins in AHF patients without and with overlapping MetS. To our knowledge, this is the first study on EL and its association with plasma lipids/lipoproteins in AHF.

Disturbed haemodynamics and related volume overload with concomitant venous congestion have been found to be associated with impaired renal function, impaired absorption of water, electrolytes, and glucose, as well as decreased plasma levels of lipids and lipoproteins in HF patients^19, 22^. Decreased lipid synthesis or decreased absorption, likely due to venous congestion may underlie the reportedly decreased lipid and lipoprotein levels in HF^19^.

We found a strong positive correlation between EL plasma levels and plasma levels of lipids and lipoproteins primarily in the no-MetS group (Table 3). Positive associations with total cholesterol, LDL-cholesterol and triglycerides are in accordance with previous findings^8^. However, a strong positive association of EL with HDL-cholesterol-, THDL-p- and SHDL-p- concentrations was in sharp contrast to previous studies reporting either no^7, 23^ or a negative association of EL with HDL-cholesterol- and HDL-p- plasma concentrations^8, 15–17^. A possible explanation for this finding might be that the observed relationship does not reflect the enzyme-substrate interaction but rather a concomitant regulation of EL- and HDL- levels into the same direction by the underlying AHF pathophysiology. Indeed, the levels of EL, HDL-cholesterol, THDL-p and SHDL-p, but not LHDL-p, were decreased by venous volume overload in the no-MetS group, in which the levels of SHDL-p were slightly affected by HF severity (Supplementary Table S2). However, partial correlation analyses revealed only a weak overall impact of venous volume overload (Supplementary Table S3) and HF severity (Supplementary Table S4) on the correlation between EL- and lipid/lipoprotein plasma levels in the no-MetS group. Observed positive correlation between EL and SHDL-p plasma levels, but no correlation with LHDL-p levels and HDL-s in the no-MetS group (Table 3) suggests a disruption of the established relationship between EL and HDL, whereby EL, by acting on HDL, converts LHDL-p into SHDL-p^16, 24^. This finding is in line with a recent report on metabolic studies in humans, suggesting that complex multiple-sized HDL-p are simultaneously secreted from liver cells, whereby extracellular HDL remodelling represents a minor contribution to HDL-class diversity^25^. Notably, the EL mass determined in the present study may not necessarily be related to EL activity, which has been shown in a recent study to be negatively correlated with HDL-cholesterol in both healthy and MetS subjects^9^. Furthermore, since EL presents mainly phospholipase activity, the association of EL levels with HDL cholesterol content and size does not exclude the catabolic role of the enzyme. In the MetS group, where plasma levels of neither EL nor LHDL-p were significantly modulated by venous volume overload, EL was significantly negatively correlated with LHDL-p (Table 3). This is in line with increased HDL particle plasma concentrations in patients with partial and complete loss-of-function mutations in the EL gene as well as in EL genetic variants exhibiting decreased EL activity^15, 16^. Although the positive correlation between EL and SHDL-p was not significant a significant negative correlation between EL plasma levels and HDL-s in the MetS group (Table 3) corroborates the established role of EL in the generation of SHDL-p^16^.

It is important to note that besides EL, LPL and hepatic lipase (HL) shape the serum lipoprotein profile in healthy subjects and MetS patients^9^. Considering the decreased LPL activity in MetS together with its role in the HDL biogenesis^26, 27^ it is possible that the negative correlation of EL and LHDL-p is, at least in part, a consequence of the decreased LPL activity in MetS patients. Similarly, the strong positive correlation between EL and triglycerides in the MetS group is most likely the reflection of the concomitant increase in EL and triglycerides, the latter due to decreased LPL activity^28^, a consequence of low-grade inflammation in MetS. A previous study revealed no association between EL levels and HDL subclasses in healthy subjects^29^. The discrepancy between those results and ours might partly be due to different methodologies used for the evaluation of HDL subclasses namely NMR-spectroscopy used in our study, and the lipoprotein precipitation method^30^ used in theirs^29^.

Previous studies showed altered SN1 lipase activities in obesity and a pronounced influence of the degree of insulin resistance on EL activity^9^. Furthermore, the association of EL plasma levels with HDL-cholesterol- and HDL-phospholipid- levels, that is present in non-obese subjects, was not observed in subjects with excess adipose tissue levels^17^. Considering the influence of adiposity on lipoprotein size and subclass concentrations^31^, as well as on EL plasma levels^17^ together with increased EL phospholipase activity in obese and insulin resistant subjects^9^, we assumed that a higher BMI and the pronounced positive correlation between EL and BMI in the MetS group (unadjusted: rho = 0.29, p = 0.008; adjusted for T2DM: rho = 0.27, p = 0.016) might, at least in part, explain the different associations between EL- and lipid/lipoprotein- plasma levels in the MetS and no-MetS groups. However, partial correlation analyses showed only a weak impact of BMI (Supplementary Table S5), waist circumference (Supplementary Table S6) and T2DM (Supplementary Table S1) on the correlation between EL- and lipid/lipoprotein plasma levels in both the no-MetS and the MetS group.

Increased EL levels and altered lipid/lipoprotein levels in MetS might be responsible for a less pronounced impact of venous volume overload on EL- and lipid/lipoprotein- levels in the MetS patients than in the no-MetS patients. The significant positive association of EL- and SLDL-p- levels and the significant negative association of EL and LDL-s in the MetS group is in accordance with the promotion of the catabolism of apoB-lipoprotein- containing lipoproteins observed in a hyperlipidaemic mouse model^32^. The results of our study regarding EL can be compared with a previous study, which revealed a positive association of HL and small, dense LDL-p^33^.

While we found no correlation between EL and IL-6 plasma levels (not shown), a significant positive correlation was established between EL and CRP, but only in the MetS group (rho: 0.24; p = 0.032). The absence of correlation in the no-MetS group could be due to the pathophysiology of the underlying volume overload, causing a pronounced significant increase in CRP (Supplementary Fig. S1) with concomitantly decreased EL (Table 4).

This study has several limitations: The insufficient quality of NMR-spectra obtained for some lipoprotein subfractions resulted in differing numbers of patients in which particular lipoprotein subfractions could be analysed. Although previous studies showed that pre- and post-heparin EL plasma levels are highly correlated^8^ or similar^34^, the possibility cannot be excluded that post-heparin EL plasma levels would be differently affected by AHF-associated pathophysiological conditions and MetS compared to our findings with pre-heparin EL plasma levels. Furthermore, considering EL polymorphisms associated with decreased EL enzyme activity^15, 16^, together with possibly altered plasma levels of EL inhibitors, such as angiopoetin-like protein 3^35^ or protein convertases^36^ in AHF, the association of EL activity with plasma lipids/lipoproteins^9^ might be different from what was found for the EL mass in this study. The study examined only the relationship between EL and HDL levels without addressing HDL functionality, a factor that is more important than HDL levels^37^, and can be studied by a variety of available experimental methods^38^. Based on our results, we conclude that the association of EL and atherogenic lipid profile is altered in AHF and additionally modified by MetS, which also strongly modulates EL- and lipid/lipoprotein- plasma levels in AHF.

## Methods

### Study design and patients

The AHF study was performed as an observational single-centre study and included consecutive white adult hospitalised AHF patients. The study was approved by the Ethics Committee of the University Hospital Centre Sisters of Charity, Zagreb, and of the Medical University of Graz. The investigation conforms with the principles outlined in the *Declaration of Helsinki* principles^39^, and informed consent was obtained in compliance with Good Clinical Practice. 152 patients were recruited from November 2013 to February 2015 as described^40^. Categorisation of the patients was performed according to the European Society of Cardiology (ESC) and ACCF/AHA Guidelines for HF^41–43^ and patients were treated by the standard ESC Guidelines for AHF^42, 43^. MetS was defined as the manifestation of three or more of the following five abnormalities: central obesity, hypertriglyceridaemia, lowered HDL-cholesterol, glucose intolerance or elevated fasting glucose and hypertension^44^. Hypertension was diagnosed according to the ESC criteria^45^. Diabetes was diagnosed in patients with dietary treatment, antidiabetic medication or current fasting plasma glucose levels higher than 7.0 mmol/L^46^. Hypercholesterolaemia was defined as LDL-cholesterol levels higher than 3.5 mmol/L, or taking a lipid-lowering drug and hypertriglyceridaemia was defined as triglyceride levels higher than 1.7 mmol/L. Patients were also classified according to three signs implying volume overload (enlarged liver, peripheral oedema or ascites) as a consequence of right-sided HF into those having none of the signs (no sign(s)) and those having at least one sign (sign(s)). Patients with severe renal failure (serum creatinine ≥400 mmol/L), renal replacement therapy, hepatic cirrhosis, malignancy, trauma, surgical diseases, pregnancy, major systemic disease or younger than 18 years were not included in the study.

### Laboratory assays

For routine laboratory assays blood was obtained at admission to the hospital. It was collected in 6 mL tubes, VACUETTE® Z Serum Clot Activator (Greiner Bio-one GmbH, Kremsmuenster, Austria). The serum aliquots were stored at −80 °C. The Beckman Coulter instrument AU 2700, 2007 (Brea, CA, USA) and Architect c8000, Abbott 2013 (Chicago, IL, USA) were used to measure total plasma cholesterol, LDL-cholesterol, HDL-cholesterol, triglycerides and CRP. A specific chemiluminescent ELISA (QuantiGlo; R&D Systems, Wiesbaden-Nordenstadt, Germany) was used to measure IL-6 concentrations and with Human Endothelial Lipase Assay Kit (TaKaRa, Takara Bio Europe S.A.S., Saint-Germain-en-Laye, France) were measured pre-heparin EL protein levels, according to the manufacturer’s instructions.

### Lipoprotein profiling by Nuclear Magnetic Resonance (NMR) spectroscopy

The lipoprotein profiles of 138 serum samples were analysed with the *AXINON* ^®^ *lipoFIT* ^®^ *-*S100 test system (Numares Health, Regensburg, Germany). NMR spectra were recorded at a temperature of 310 K on a shielded 600 MHz Bruker Avance III HD spectrometer as described^47^. To ensure data quality only spectra meeting defined quality criteria were used for analyses. The number (n) of analysed samples is indicated in Table 2.

### Statistical analysis

Categorical data are shown as absolute and relative frequencies, and continuous data are presented as mean and SD or as median and range (minimum to maximum) depending on distribution. The differences between patients with and without metabolic syndrome were assessed by t-test or Mann-Whitney U test for continuous parameters and by Fisher’s exact test for categorical parameters. In addition, the differences between patients with and without sign(s) implying volume overload were assessed within the groups using Mann-Whitney U test. The Spearman correlation coefficients were calculated to evaluate the correlation of EL plasma levels and laboratory parameters. In addition, the differences between patients with and without sign(s) implying venous volume overload (enlarged liver, peripheral oedema or ascites), were assessed within the metabolic syndrome groups using Mann-Whitney U test. Furthermore, the impact of T2DM, sign(s) implying venous volume overload, BMI, waist circumference and NYHA classes (2/3 vs. 4) on the correlations between EL and lipid parameters was assessed by means of partial correlations. A p-value < 0.05 was considered statistically significant and results are to be interpreted in an exploratory fashion. All data were analysed using R version 3.3.1.

## Electronic supplementary material

Supplementary Information
