# Supplementary material for: Metabolic Syndrome Modulates Association between Endothelial Lipase and Lipid/Lipoprotein Plasma Levels in Acute Heart Failure Patients
Source: Sci Rep. 2017 Apr 26;7:1165. doi: 10.1038/s41598-017-01367-2 (PMC5430647; doi:10.1038/s41598-017-01367-2)
Supplement: Supplementary file 1 — Supplementary Information [file 41598_2017_1367_MOESM1_ESM.pdf]

Metabolic Syndrome Modulates Association between Endothelial Lipase and Lipid/Lipoprotein Plasma Levels in Acute Heart Failure Patients

Ines Potočnjak, Matias Trbušić, Sanda Dokoza Terešak, Bojana Radulović, Gudrun Pregartner, Andrea Berghold, Beate Tiran, Gunther Marsche, Vesna Degoricija, and Saša Frank

Supplementary Table S1. Impact of T2DM on Spearman correlation between EL- and lipid/lipoprotein- levels (partial correlations)

	**no-MetS**		**MetS**	
	**rho**	**p-value**	**rho**	**p-value**
Total cholesterol (mmol/L)	**0.45**	**<0.001**	0.20	0.070
LDL-cholesterol (mmol/L)	**0.36**	**0.003**	0.18	0.105
HDL-cholesterol (mmol/L)	**0.43**	**<0.001**	-0.11	0.334
Triglycerides (mmol/L)	**0.32**	**0.011**	0.29	**0.009**
LVLDL-p (nmol/L)	0.20	0.307	0.11	0.458
TLDL-p (nmol/L)	**0.26**	**0.048**	0.18	0.118
LLDL-p (mmol/L)	0.22	0.103	-0.01	0.964
SLDL-p (nmol/L)	0.03	0.825	0.24	**0.040**
THDL-p (nmol/L)	**0.48**	**<0.001**	0.16	0.160
LHDL-p (nmol/L)	0.09	0.516	**-0.39**	**0.003**
SHDL-p (nmol/L)	**0.38**	**0.010**	0.22	0.080
VLDL-s (nm)	0.14	0.288	-0.11	0.341
LDL-s (nm)	0.12	0.362	-0.22	0.063
HDL-s (nm)	-0.16	0.222	**-0.32**	**0.006**

HDL-high density lipoprotein; LDL-low-density lipoprotein; LVLDL-p –large very low-density lipoprotein particle; TLDL-p –total LDL particles; LLDL-p –large LDL particles; SLDL-p –small LDL-p; THDL-p –total HDL-particles; LHDL-p –large HDL-p; SHDL-p –small HDL-p; VLDL-s –VLDL size; LDL-s –LDL size; HDL-s –HDL size. Significant associations are depicted in bold.

Supplementary Table S2. EL- and lipid/lipoprotein-levels in different NYHA classes

No-MetS			
**Variable**	**NYHA 2/3**	**NYHA 4**	**P-value**
EL (pg/mL)	354.0 [74.1-1013.4]	465.0 [77.8-704.3]	0.183
Total cholesterol (mmol/L)	3.7 [2.2-9.1]	4.3 [2.3-8.5]	0.072
LDL-cholesterol (mmol/L)	2.3 [0.8-6.3]	2.6 [1.4-6.1]	0.270
HDL-cholesterol (mmol/L)	1.0 [0.5-1.6]	1.1 [0.5-2.3]	0.077
Triglycerides (mmol/L)	0.9 [0.6-3.0]	1.0 [0.5-1.8]	0.264
LVLDL-p (nmol/L)	1.8 [1.5-3.7]	2.0 [1.6-5.1]	0.492
TLDL-p (nmol/L)	1097 [615-1912]	1104 [564-2488]	0.239
LLDL-p (nmol/L)	630.5 [304-1379]	709 [311-2078]	0.282
SLDL-p (nmol/L)	454.5 [176-1440]	442.5 [225-1082]	0.961
THDL-p (nmol/L)	20926 [3711-37506]	24182 [8638-34795]	0.138
LHDL-p (nmol/L)	6317.5 [2943-11541]	6176 [4220-14726]	0.588
SHDL-p (nmol/L)	15486.5 [7086-31995]	18460 [10463-32390]	**0.045**
VLDL-s (nm)	47.1 [41.0-52.3]	47.1 [39.4-51.5]	0.659
LDL-s (nm)	21.3 [20.8-22.0]	21.3 [20.7-22.7]	0.623
HDL-s (nm)	9.3 [8.5-10.3]	9.4 [8.5-10.5]	0.761
**MetS**			
**Variable**	**NYHA 2/3**	**NYHA 4**	**P-value**
EL (pg/mL)	455.1 [157.6-1407.7]	511.3 [221.9-1371.5]	0.546
Total cholesterol (mmol/L)	3.8 [1.7-7.7]	4.3 [2.4-6.4]	0.932
LDL-cholesterol (mmol/L)	2.3 [1.0-6.0]	2.3 [1.2-4.4]	0.950
HDL-cholesterol (mmol/L)	0.9 [0.3-1.8]	0.9 [0.4-3.6]	0.820
Triglycerides (mmol/L)	1.1 [0.6-4.3]	1.3 [0.7-2.4]	0.222
LVLDL-p (nmol/L)	2.7 [1.5-10.6]	2.8 [1.6-8.3]	0.868
TLDL-p (nmol/L)	1093.5 [360-2425]	1147 [614-1857]	0.965
LLDL-p (nmol/L)	663.5 [306-1160]	708 [300-1089]	0.761
SLDL-p (nmol/L)	476.5 [172-1331]	468.5 [172-828]	0.959
THDL-p (nmol/L)	20864 [7910-36951]	18329 [8493-33358]	0.411
LHDL-p (nmol/L)	5324 [2835-14921]	4500 [2829-9207]	0.078
SHDL-p (nmol/L)	16502 [6332-34210]	16751.5 [6352-27078]	0.563
VLDL-s (nm)	47.2 [37.1-51.3]	46.7 [41.1-50.9]	0.487
LDL-s (nm)	21.1 [20.2-21.8]	21.2 [20.7-21.9]	0.673
HDL-s (nm)	9.1 [8.3-10.6]	9.0 [8.3-9.8]	0.864

Results are presented as median, minimum and maximum. Differences between NYHA classes 2/3 and 4 were assessed by Mann-Whitney U-test; significant differences are depicted in bold. HDL-high density lipoprotein; LDL-low-density lipoprotein; EL-endothelial lipase; LVLDL-p –large very low-density lipoprotein particles; TLDL-p –total LDL particles; LLDL-p –large LDL particles; SLDL-p –small LDL-p; THDL-p –total HDL-particles; LHDL-p –large HDL-p; SHDL-p –small HDL-p; VLDL-s –VLDL size; LDL-s –LDL size; HDL-s –HDL size

Supplementary Table S3. Impact of sign(s) implying volume overload on Spearman correlation between EL- and lipid/lipoprotein- levels (partial correlation)

	**no-MetS**		**MetS**	
	**rho**	**p-value**	**rho**	**p-value**
Total cholesterol (mmol/L)	0.40	**0.001**	0.16	0.155
LDL-cholesterol (mmol/L)	0.32	**0.010**	0.13	0.249
HDL-cholesterol (mmol/L)	0.39	**0.002**	-0.13	0.246
Triglycerides (mmol/L)	0.24	0.052	0.27	**0.016**
LVLDL-p (nmol/L)	0.11	0.584	0.11	0.464
TLDL-p (nmol/L)	0.25	0.061	0.14	0.222
LLDL-p (mmol/L)	0.18	0.170	-0.04	0.737
SLDL-p (nmol/L)	0.13	0.335	0.22	0.064
THDL-p (nmol/L)	0.42	**0.001**	0.14	0.247
LHDL-p (nmol/L)	0.12	0.381	-0.37	**0.006**
SHDL-p (nmol/L)	0.22	0.138	0.21	0.097
VLDL-s (nm)	0.22	0.089	-0.12	0.309
LDL-s (nm)	0.05	0.706	-0.23	0.053
HDL-s (nm)	-0.05	0.681	-0.30	**0.010**

HDL-high density lipoprotein; LDL-low-density lipoprotein; LVLDL-p –large very low-density lipoprotein particle; TLDL-p –total LDL particles; LLDL-p –large LDL particles; SLDL-p –small LDL-p; THDL-p –total HDL-particles; LHDL-p –large HDL-p; SHDL-p –small HDL-p; VLDL-s –VLDL size; LDL-s –LDL size; HDL-s –HDL size. Significant associations are depicted in bold.

Supplementary Table S4. Impact of NYHA classes on Spearman correlation between EL- and lipid/lipoprotein- levels (partial correlation)

	no-MetS		MetS	
	**rho**	**p-value**	**rho**	**p-value**
Total cholesterol (mmol/L)	0.44	**<0.001**	0.19	0.092
LDL-cholesterol (mmol/L)	0.36	**0.003**	0.16	0.154
HDL-cholesterol (mmol/L)	0.43	**<0.001**	-0.11	0.321
Triglycerides (mmol/L)	0.28	**0.022**	0.29	**0.009**
LVLDL-p (nmol/L)	0.18	0.357	0.12	0.439
TLDL-p (nmol/L)	0.25	0.051	0.16	0.162
LLDL-p (mmol/L)	0.21	0.110	-0.02	0.870
SLDL-p (nmol/L)	0.06	0.652	0.24	**0.043**
THDL-p (nmol/L)	0.45	**<0.001**	0.17	0.138
LHDL-p (nmol/L)	0.12	0.404	-0.39	**0.004**
SHDL-p (nmol/L)	0.27	0.069	0.23	0.067
VLDL-s (nm)	0.16	0.227	-0.12	0.327
LDL-s (nm)	0.08	0.524	-0.25	**0.034**
HDL-s (nm)	-0.14	0.303	-0.32	**0.006**

HDL-high density lipoprotein; LDL-low-density lipoprotein; LVLDL-p –large very low-density lipoprotein particle; TLDL-p –total LDL particles; LLDL-p –large LDL particles; SLDL-p –small LDL-p; THDL-p –total HDL-particles; LHDL-p –large HDL-p; SHDL-p –small HDL-p; VLDL-s –VLDL size; LDL-s –LDL size; HDL-s –HDL size. Significant associations are depicted in bold.

Supplementary Table S5. Impact of BMI on Spearman correlation between EL- and lipid/lipoprotein- levels (partial correlation)

	no-MetS		MetS	
	**rho**	**p-value**	**rho**	**p-value**
Total cholesterol (mmol/L)	**0.49**	**<0.001**	0.21	0.066
LDL-cholesterol (mmol/L)	**0.40**	**0.001**	0.17	0.119
HDL-cholesterol (mmol/L)	**0.50**	**<0.001**	-0.07	0.552
Triglycerides (mmol/L)	**0.31**	**0.013**	**0.27**	**0.016**
LVLDL-p (nmol/L)	0.25	0.195	0.06	0.676
TLDL-p (nmol/L)	**0.30**	**0.023**	0.16	0.171
LLDL-p (mmol/L)	**0.27**	**0.044**	0.00	0.970
SLDL-p (nmol/L)	0.07	0.594	**0.25**	**0.032**
THDL-p (nmol/L)	**0.55**	**<0.001**	0.12	0.303
LHDL-p (nmol/L)	0.16	0.259	**-0.34**	**0.012**
SHDL-p (nmol/L)	**0.43**	**0.003**	0.17	0.161
VLDL-s (nm)	0.12	0.353	-0.06	0.620
LDL-s (nm)	0.08	0.554	**-0.24**	**0.037**
HDL-s (nm)	-0.15	0.267	**-0.23**	**0.049**

HDL-high density lipoprotein; LDL-low-density lipoprotein; LVLDL-p –large very low-density lipoprotein particle; TLDL-p –total LDL particles; LLDL-p –large LDL particles; SLDL-p –small LDL-p; THDL-p –total HDL-particles; LHDL-p –large HDL-p; SHDL-p –small HDL-p; VLDL-s –VLDL size; LDL-s –LDL size; HDL-s –HDL size. Significant associations are depicted in bold.

Supplementary Table S6. Impact of waist circumference on Spearman correlation between EL- and lipid/lipoprotein- levels (partial correlation)

	**no-MetS**		**MetS**	
	**rho**	**p-value**	**rho**	**p-value**
Total cholesterol (mmol/L)	0.48	**<0.001**	0.19	0.090
LDL-cholesterol (mmol/L)	0.40	**0.001**	0.16	0.154
HDL-cholesterol (mmol/L)	0.48	**<0.001**	-0.11	0.313
Triglycerides (mmol/L)	0.32	**0.011**	0.29	**0.008**
LVLDL-p (nmol/L)	0.19	0.346	0.08	0.597
TLDL-p (nmol/L)	0.28	**0.031**	0.17	0.159
LLDL-p (mmol/L)	0.25	0.057	-0.01	0.940
SLDL-p (nmol/L)	0.07	0.597	0.24	**0.041**
THDL-p (nmol/L)	0.51	**<0.001**	0.17	0.156
LHDL-p (nmol/L)	0.10	0.476	-0.38	**0.004**
SHDL-p (nmol/L)	0.37	**0.011**	0.22	0.079
VLDL-s (nm)	0.13	0.321	-0.11	0.353
LDL-s (nm)	0.11	0.413	-0.24	**0.037**
HDL-s (nm)	-0.16	0.215	-0.31	**0.007**

HDL-high density lipoprotein; LDL-low-density lipoprotein; LVLDL-p –large very low-density lipoprotein particle; TLDL-p –total LDL particles; LLDL-p –large LDL particles; SLDL-p –small LDL-p; THDL-p –total HDL-particles; LHDL-p –large HDL-p; SHDL-p –small HDL-p; VLDL-s –VLDL size; LDL-s –LDL size; HDL-s –HDL size. Significant associations are depicted in bold.

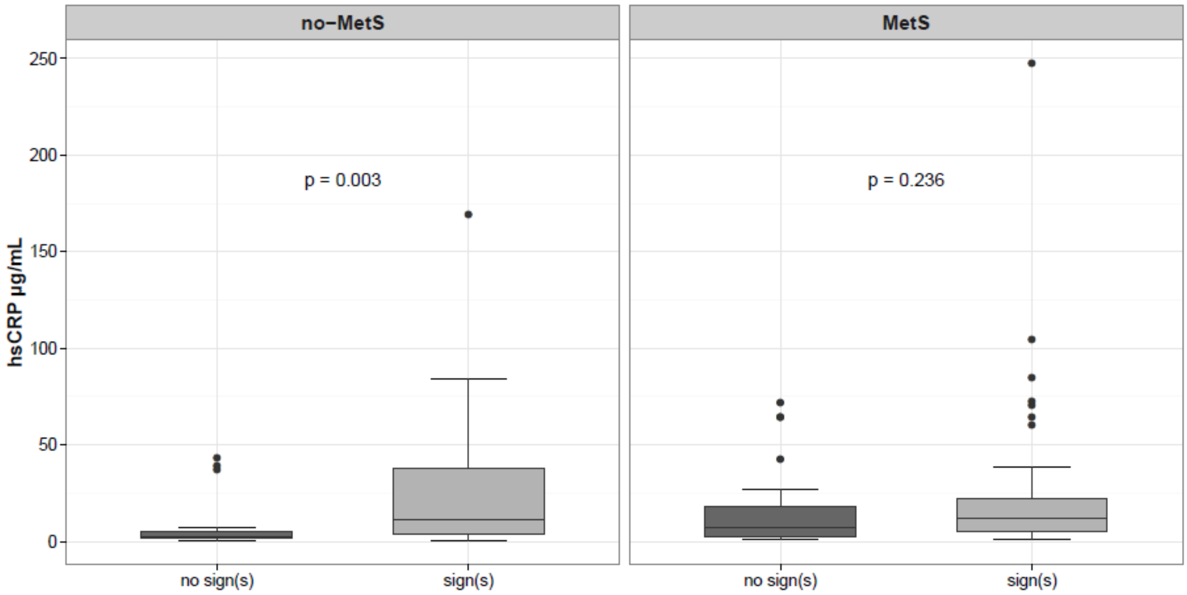

Supplementary Fig. S1. CRP plasma levels in the study group: CRP protein levels were measured in the serum of 18 no-MetS patients with no sign(s) and of 47 no-MetS patients with sign(s) implying volume overload as well in the serum of 24 MetS patients with no sign(s) and of 61 MetS patients with sign(s) implying volume overload. The box corresponds to the first and third quartiles, the horizontal line to the median and the whiskers of the plot extend to the highest or lowest value that lies within 1.5*IQR of the respective quartile; outliers are represented as individual dots.
